# Supplementary material for: Inflammatory and nutritional indexes as predictors of acute kidney injury in patients with Immunoglobulin A nephropathy: a retrospective study
Source: PeerJ. 2025 Aug 20;13:e19917. doi: 10.7717/peerj.19917 (PMC12374690; doi:10.7717/peerj.19917)
Supplement: Supplemental Information 1 — Full model: NS, T, eGFR, BUN, 24h-UPRO, PNI, CRP, SIRI, LMR, PLR, and LCR; Model 1: NS, T, eGFR, BUN, 24h-UPRO, PNI and CRP; Model 2: NS, T, eGFR, BUN, 24h-UPRO, PNI and SIRI; Model 3: NS, T, eGFR, BUN, 24h-UPRO, PNI and LMR; Model 4: NS, T, eGFR, BUN, 24h-UPRO, PNI and PLR; Model 5: NS, T, eGFR, BUN, 24h-UPRO, PNI and LCR; Model 6: eGFR, BUN and CRP. NS, nephrotic syndrome; T, interstitial fibrosis/tubular atrophy; eGFR, estimated glomerular filtration rate; BUN, blood urea nitrogen; 24h-UPRO, 24-hour urinary protein quantification; PNI, prognostic nutritional index; CRP, C-reactive protein; SIRI, systemic inflammation response index; LMR, lymphocyte to monocyte ratio; PLR, platelet to lymphocyte ratio; LCR, lymphocyte to C-reactive protein ratio. [file peerj-13-19917-s001.docx]

Supplementary Table S1. The P values from the Hosmer-Lemeshow test for the seven models in both the training and testing datasets.

|  | **P value for Hosmer-Lemeshow test** | |
| --- | --- | --- |
|  | **Training Set** | **Test Set** |
| **Full model** | 0.764 | 0.261 |
| **Model 1** | 0.326 | 0.119 |
| **Model 2** | 0.518 | 0.365 |
| **Model 3** | 0.087 | 0.296 |
| **Model 4** | 0.161 | 0.527 |
| **Model 5** | 0.467 | **0.002** |
| **Model 6** | 0.131 | 0.499 |

Full model: NS, T, eGFR, BUN, 24h-UPRO, PNI, CRP, SIRI, LMR, PLR, and LCR; Model 1: NS, T, eGFR, BUN, 24h-UPRO, PNI and CRP; Model 2: NS, T, eGFR, BUN, 24h-UPRO, PNI and SIRI; Model 3: NS, T, eGFR, BUN, 24h-UPRO, PNI and LMR; Model 4: NS, T, eGFR, BUN, 24h-UPRO, PNI and PLR; Model 5: NS, T, eGFR, BUN, 24h-UPRO, PNI and LCR; Model 6: eGFR, BUN and CRP. NS, nephrotic syndrome; T, interstitial fibrosis/tubular atrophy; eGFR, estimated glomerular filtration rate; BUN, blood urea nitrogen; 24h-UPRO, 24-hour urinary protein quantification; PNI, prognostic nutritional index; CRP, C-reactive protein; SIRI, systemic inflammation response index; LMR, lymphocyte to monocyte ratio; PLR, platelet to lymphocyte ratio; LCR, lymphocyte to C-reactive protein ratio.
