# Supplementary material for: Inflammatory and nutritional indexes as predictors of acute kidney injury in patients with Immunoglobulin A nephropathy: a retrospective study
Source: PeerJ. 2025 Aug 20;13:e19917. doi: 10.7717/peerj.19917 (PMC12374690; doi:10.7717/peerj.19917)
Supplement: Supplemental Information 2 — AUC, area under the curve; AKI, acute kidney injury; IgAN, Immunoglobulin A nephropathy; LASSO, least absolute shrinkage and selection operator; T, interstitial fibrosis/tubular atrophy; eGFR, estimated glomerular filtration rate; BUN, blood urea nitrogen; 24h-UPRO, 24-hour urinary protein quantification; PNI, prognostic nutritional index; CRP, C-reactive protein; SIRI, systemic inflammation response index; LMR, lymphocyte to monocyte ratio; PLR, platelet to lymphocyte ratio; LCR, lymphocyte to C-reactive protein ratio. [file peerj-13-19917-s002.docx]

Supplementary Table S1. The AUC values of composite indices and their components for AKI prediction in IgAN patients among the training set.

| **Index** | **AUC (95%CI)** |
| --- | --- |
| **PNI** | 0.597 (0.513-0.682) |
| albumin | 0.574 (0.489-0.659) |
| lymphocyte | 0.618 (0.524-0.712) |
| **SIRI** | 0.656 (0.585-0.726) |
| neutrophil | 0.609 (0.523-0.695) |
| monocyte | 0.558 (0.477-0.640) |
| lymphocyte | 0.618 (0.524-0.712) |
| **LMR** | 0.666 (0.591-0.741) |
| lymphocyte | 0.618 (0.524-0.712) |
| monocyte | 0.558 (0.477-0.640) |
| **PLR** | 0.581 (0.488-0.674) |
| platelet | 0.544 (0.456-0.631) |
| lymphocyte | 0.618 (0.524-0.712) |
| **LCR** | 0.684 (0.604-0.764) |
| lymphocyte | 0.618 (0.524-0.712) |
| CRP | 0.659 (0.577-0.741) |

AUC, area under the curve; AKI, acute kidney injury; IgAN, Immunoglobulin A nephropathy; LASSO, least absolute shrinkage and selection operator; T, interstitial fibrosis/tubular atrophy; eGFR, estimated glomerular filtration rate; BUN, blood urea nitrogen; 24h-UPRO, 24-hour urinary protein quantification; PNI, prognostic nutritional index; CRP, C-reactive protein; SIRI, systemic inflammation response index; LMR, lymphocyte to monocyte ratio; PLR, platelet to lymphocyte ratio; LCR, lymphocyte to C-reactive protein ratio.
