# Supplementary figures and images for: Inflammatory and nutritional indexes as predictors of acute kidney injury in patients with Immunoglobulin A nephropathy: a retrospective study
Source: PeerJ. 2025 Aug 20;13:e19917. doi: 10.7717/peerj.19917 (PMC12374690; doi:10.7717/peerj.19917)

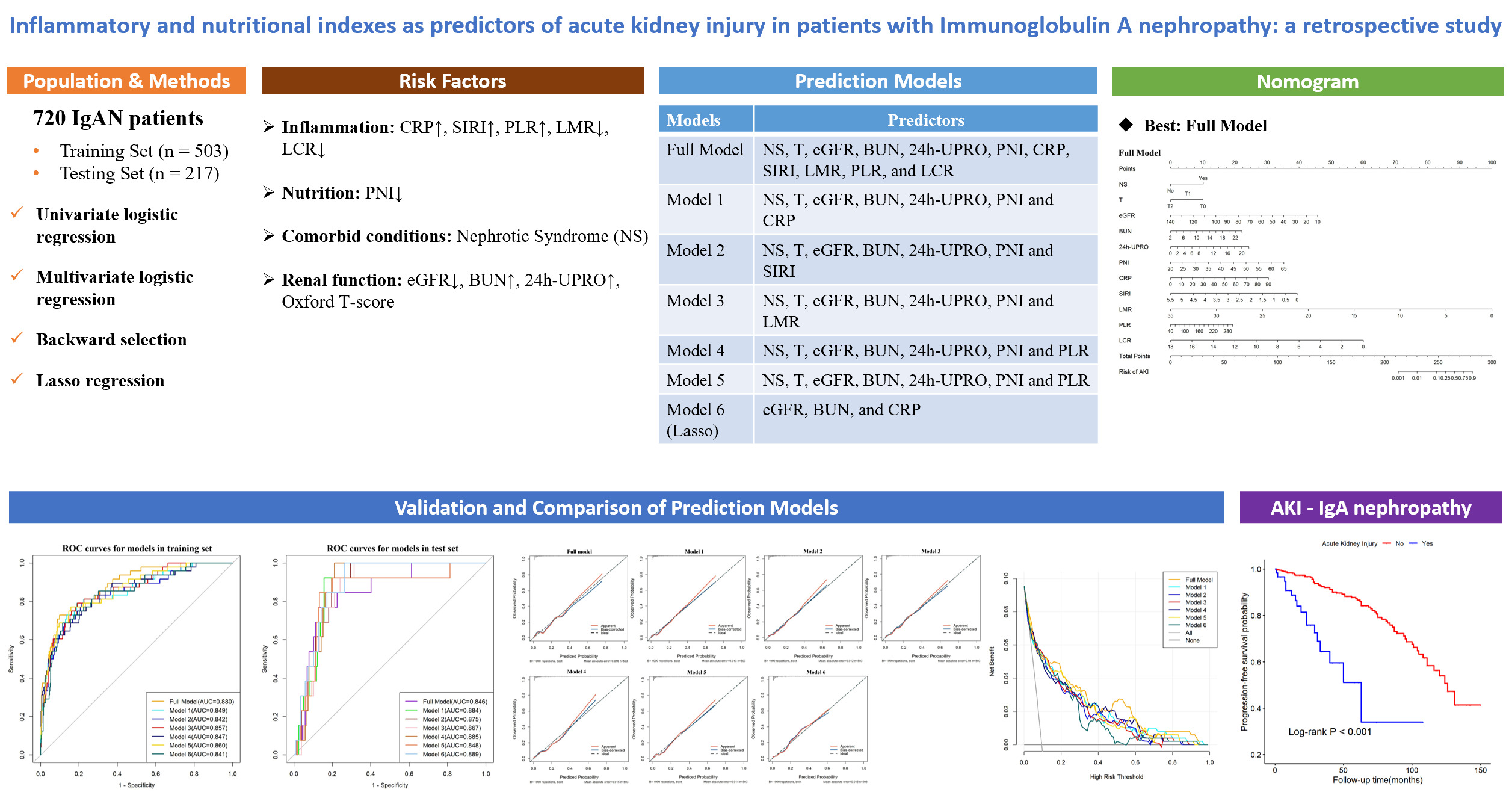

Supplement: Supplemental Information 3 — AKI, acute kidney injury; IgAN, Immunoglobulin A nephropathy; LASSO, least absolute shrinkage and selection operator; T, interstitial fibrosis/tubular atrophy; eGFR, estimated glomerular filtration rate; BUN, blood urea nitrogen; 24h-UPRO, 24-hour urinary protein quantification; PNI, prognostic nutritional index; CRP, C-reactive protein; SIRI, systemic inflammation response index; LMR, lymphocyte to monocyte ratio; PLR, platelet to lymphocyte ratio; LCR, lymphocyte to C-reactive protein ratio. [file peerj-13-19917-s003.png]

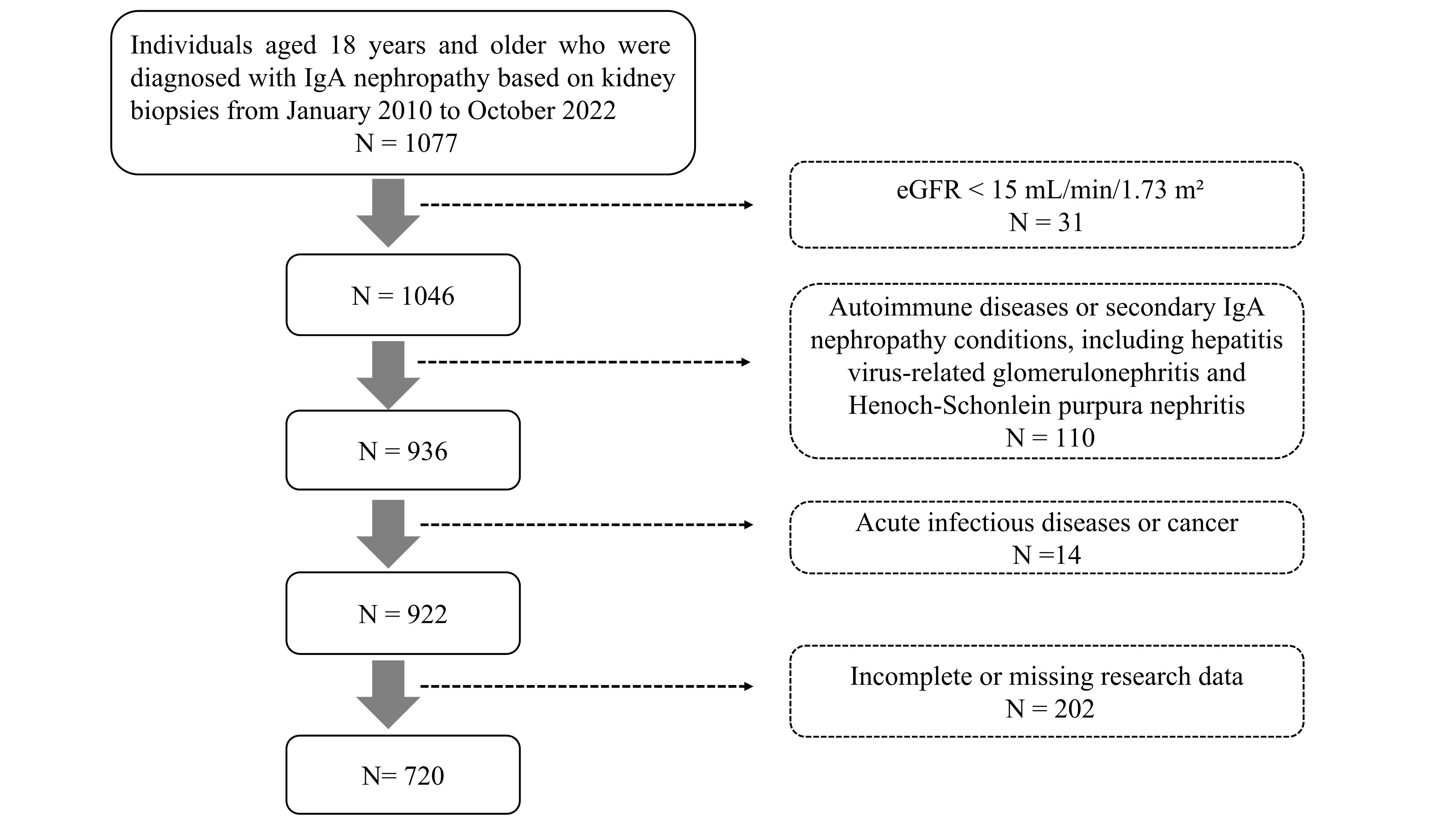

Supplement: Supplemental Information 4 [file peerj-13-19917-s004.png]

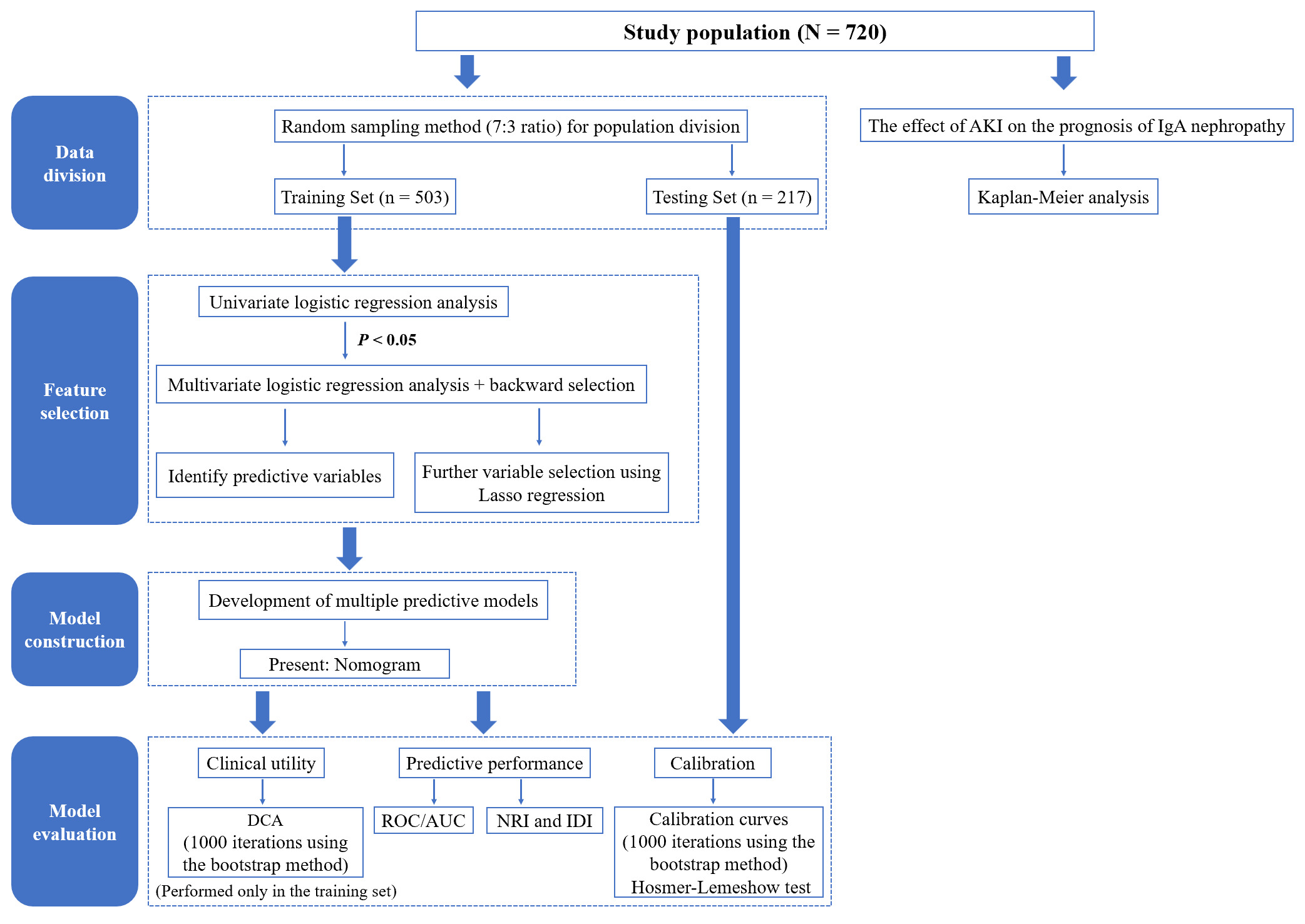

Supplement: Supplemental Information 5 — AKI, acute kidney injury; IgAN, Immunoglobulin A nephropathy; LASSO, least absolute shrinkage and selection operator; DCA, decision curve analysis; ROC, receiver operating characteristic; AUC, area under the curve; NRI, net reclassification improvement; IDI, net reclassification improvement. [file peerj-13-19917-s005.png]

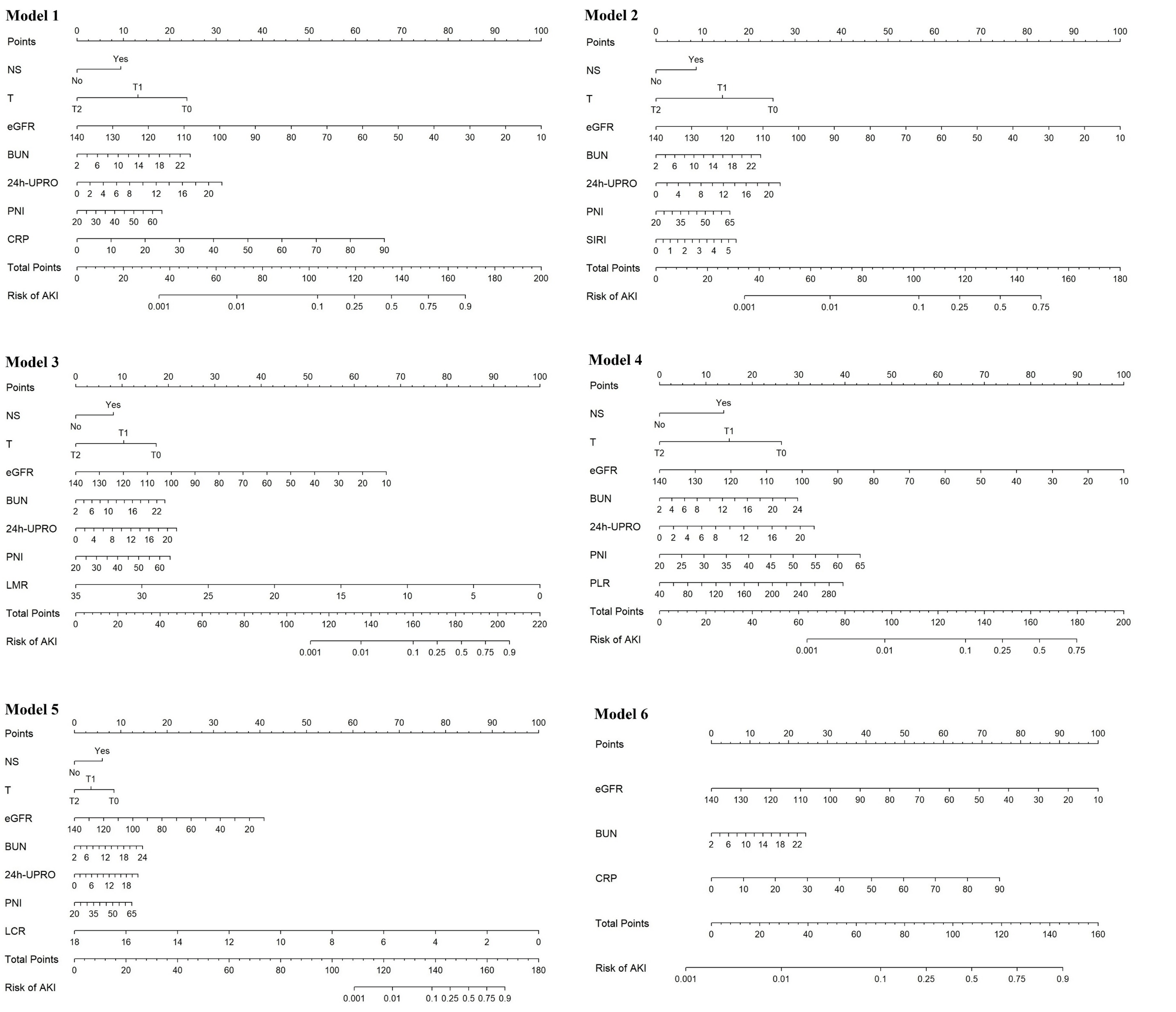

Supplement: Supplemental Information 6 — AKI, acute kidney injury; IgAN, Immunoglobulin A nephropathy; NS, nephrotic syndrome; T, interstitial fibrosis/tubular atrophy; eGFR, estimated glomerular filtration rate; BUN, blood urea nitrogen; 24h-UPRO, 24-hour urinary protein quantification; PNI, prognostic nutritional index; CRP, C-reactive protein; SIRI, systemic inflammation response index; LMR, lymphocyte to monocyte ratio; PLR, platelet to lymphocyte ratio; LCR, lymphocyte to C-reactive protein ratio. [file peerj-13-19917-s006.png]

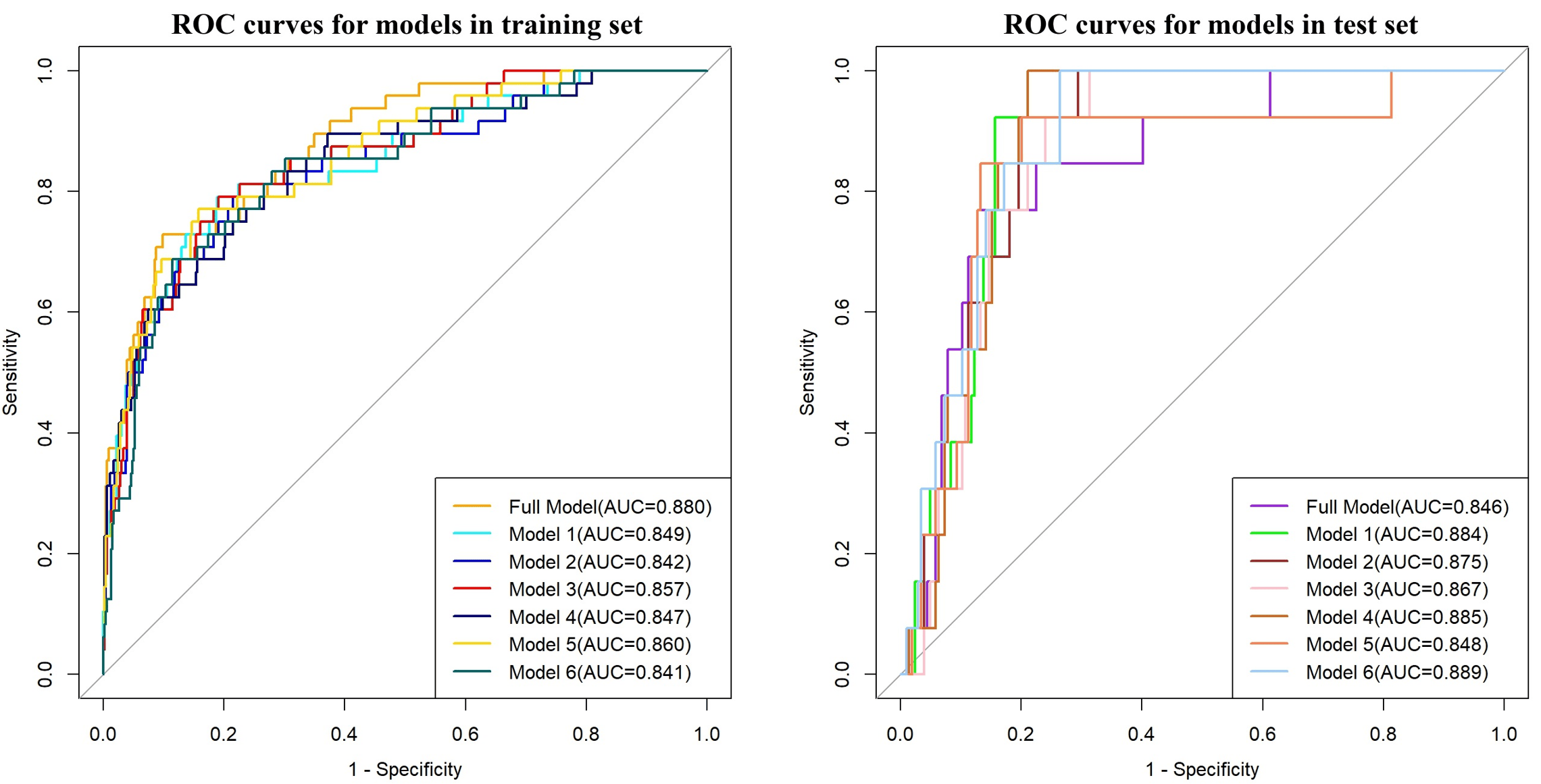

Supplement: Supplemental Information 7 — Full model: NS, T, eGFR, BUN, 24h-UPRO, PNI, CRP, SIRI, LMR, PLR, and LCR; Model 1: NS, T, eGFR, BUN, 24h-UPRO, PNI and CRP; Model 2: NS, T, eGFR, BUN, 24h-UPRO, PNI and SIRI; Model 3: NS, T, eGFR, BUN, 24h-UPRO, PNI and LMR; Model 4: NS, T, eGFR, BUN, 24h-UPRO, PNI and PLR; Model 5: NS, T, eGFR, BUN, 24h-UPRO, PNI and LCR; Model 6: eGFR, BUN and CRP. NS, nephrotic syndrome; T, interstitial fibrosis/tubular atrophy; eGFR, estimated glomerular filtration rate; BUN, blood urea nitrogen; 24h-UPRO, 24-hour urinary protein quantification; PNI, prognostic nutritional index; CRP, C-reactive protein; SIRI, systemic inflammation response index; LMR, lymphocyte to monocyte ratio; PLR, platelet to lymphocyte ratio; LCR, lymphocyte to C-reactive protein ratio. [file peerj-13-19917-s007.png]

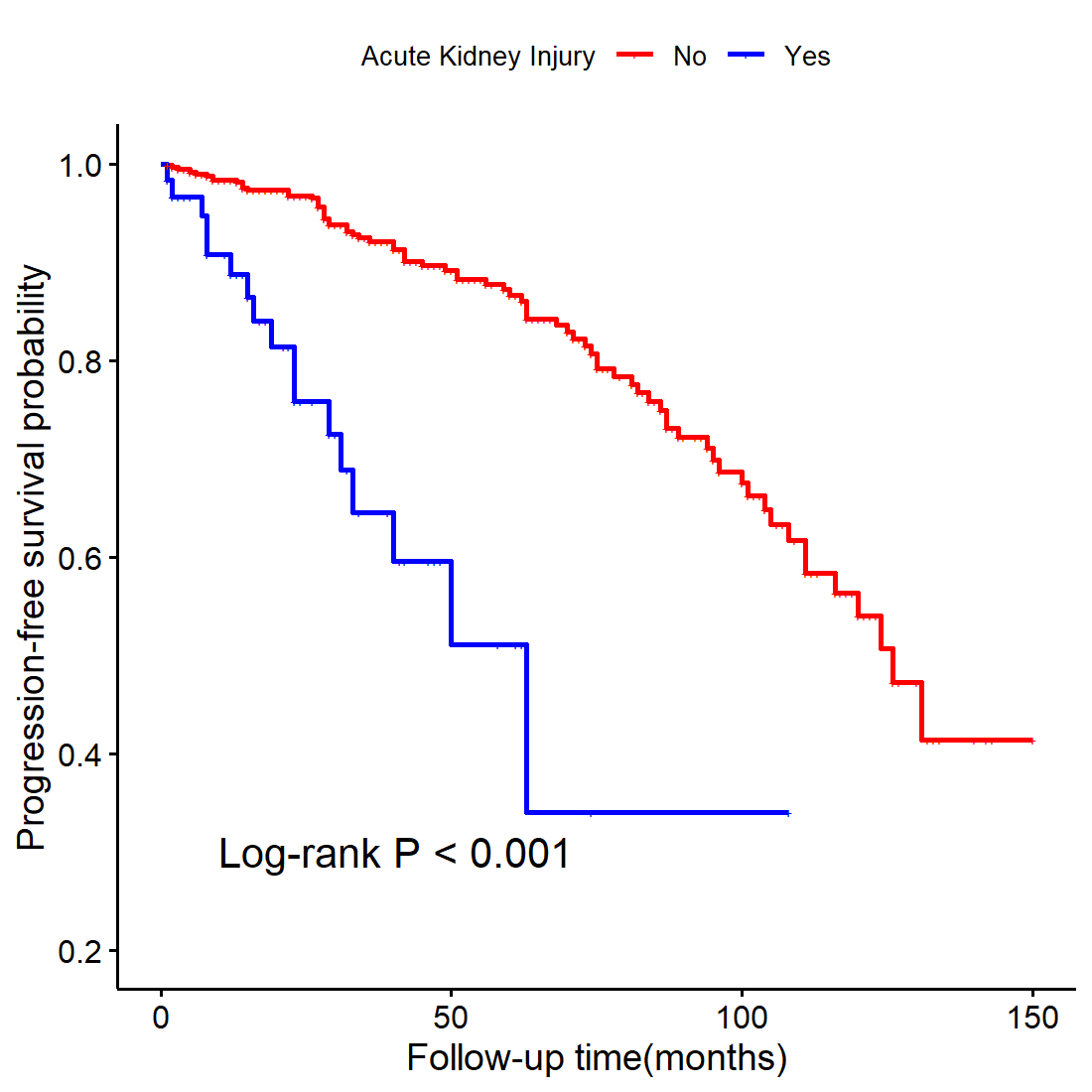

Supplement: Supplemental Information 8 — IgAN, Immunoglobulin A nephropathy; AKI, acute kidney injury. [file peerj-13-19917-s008.png]
