# Supplementary material for: Inflammatory and nutritional indexes as predictors of acute kidney injury in patients with Immunoglobulin A nephropathy: a retrospective study
Source: PeerJ. 2025 Aug 20;13:e19917. doi: 10.7717/peerj.19917 (PMC12374690; doi:10.7717/peerj.19917)
Supplement: Supplemental Information 9 [file peerj-13-19917-s009.docx]

STROBE Statement—checklist of items that should be included in reports of observational studies

|  | Item No. | Recommendation | Page  No. | Relevant text from manuscript |
| --- | --- | --- | --- | --- |
| **Title and abstract** | 1 | (*a*) Indicate the study’s design with a commonly used term in the title or the abstract | 1 | Inflammatory and nutritional indexes as predictors of acute kidney injury in patients with Immunoglobulin A nephropathy |
|  |  | (*b*) Provide in the abstract an informative and balanced summary of what was done and what was found | 1-2 | Line 13-37 of the manuscript states, “Background. Immunoglobulin A nephropathy (IgAN) patients with acute kidney injury (AKI) have elevated risk of adverse events and mortality. However, there is currently a lack of convenient and effective clinical tools to predict AKI risk in this population. The present study was conducted to creating such tools containing inflammatory and nutritional indexes. Method. Data from 720 adults diagnosed with IgAN by renal biopsy at the First Hospital of Jilin University were collected. They were randomly divided into a training set (n = 503) and a test set (n = 217) in a 7:3 ratio. Univariate and multivariate logistic regression analyses with backward selection were used to identify risk factors, resulting in multiple prediction models. Least absolute shrinkage and selection operator (LASSO) regression was used to simplify the model. The models were presented using nomograms, and their performances were evaluated through receiver operating characteristic (ROC) curves, area under the curve (AUC), Hosmer-Lemeshow test, net reclassification improvement (NRI), integrated discrimination improvement (IDI), calibration curves, and clinical decision curve analysis (DCA). Results. Eleven risk factors related to IgAN with AKI were identified, including nephrotic syndrome (NS), T score from the Oxford histological classification, estimated glomerular filtration rate (eGFR), blood urea nitrogen (BUN), 24-hour urinary protein quantification (24h-UPRO), C-reactive protein (CRP), systemic inflammatory response index (SIRI), lymphocyte-to-monocyte ratio (LMR), platelet-to-lymphocyte ratio (PLR), lymphocyte-to-CRP ratio (LCR), and prognostic nutritional index (PNI). These factors contributed to the development of seven prediction models. ROC curves indicated good predictive performance for all models, with the full model performing best. The Hosmer-Lemeshow test showed that six models, fit well in the test set. DCA results demonstrated significant clinical benefits for all models. Conclusion. This study developed and validated a series of novel and convenient AKI risk prediction models for patients with IgAN, highlighting the importance of inflammatory status and nutritional condition in assessing AKI risk.” |
| Introduction | | | |  |
| Background/rationale | 2 | Explain the scientific background and rationale for the investigation being reported | 2-3 | Lines 39 to 83 of the manuscript elaborate in detail on IgA nephropathy, acute kidney injury (AKI), and the relationship between them, and analyze the current state of research. It points out that the current research is flawed in that it fails to provide a convenient and effective clinical tool for rapidly identifying high - risk patients with IgA nephropathy complicated by AKI. In addition, it also explores the relatively new risk factors in current research. |
| Objectives | 3 | State specific objectives, including any prespecified hypotheses | 3 | The present study was conducted to identify the new risk factors of AKI in IgAN patients and creating clinical tools for AKI prediction. |
| Methods | | | |  |
| Study design | 4 | Present key elements of study design early in the paper | 3 | Located on lines 88-89 of the manuscript: " We conducted a retrospective analysis of adults (age ≥ 18 years) diagnosed with IgAN through kidney biopsy at the First Hospital of Jilin University from January 2010 to October 2022." This content clarifies that the study design is a retrospective analysis, elaborates on the selection criteria of the research subjects (adults diagnosed with IgAN through kidney biopsy at the First Hospital of Jilin University and aged ≥ 18 years), and presents the time frame of the study (from January 2010 to October 2022), preliminarily presenting the key elements of the study design. |
| Setting | 5 | Describe the setting, locations, and relevant dates, including periods of recruitment, exposure, follow-up, and data collection | 3-4 and 6 | Located on lines 88 - 89 of the manuscript: “We conducted a retrospective analysis of adults (age ≥ 18 years) diagnosed with IgAN through kidney biopsy at the First Hospital of Jilin University from January 2010 to October 2022.” This part clearly defines the research location as the First Hospital of Jilin University, and the data collection period (which is equivalent to the exposure time in a retrospective study) is from January 2010 to October 2022. Since this is a retrospective study, there is no prospective recruitment period. Instead, eligible adult IgAN patients (aged ≥ 18 years and diagnosed with IgAN through kidney biopsy) were selected from existing medical records.  Located on line 153 of the manuscript: “Follow-up time was reported in months.” This indicates that the unit of measurement for the follow-up time is months, and the follow-up starts from the time when patients were diagnosed with IgAN (during the period from January 2010 to October 2022).  Located on lines 206 - 207 of the manuscript: “The median follow-up time of participants was 26.00 months.” This supplements crucial information, determining that the median follow-up duration of the participants is 26.00 months. Overall, this study was conducted at the First Hospital of Jilin University, focusing on IgAN patients diagnosed from January 2010 to October 2022. It has clearly defined the research location, the scope of data collection time, the unit of measurement for follow-up time, and the median follow-up duration, which are all related to the research setting. |
| Participants | 6 | (*a*) *Cohort study*—Give the eligibility criteria, and the sources and methods of selection of participants. Describe methods of follow-up  *Case-control study*—Give the eligibility criteria, and the sources and methods of case ascertainment and control selection. Give the rationale for the choice of cases and controls  *Cross-sectional study*—Give the eligibility criteria, and the sources and methods of selection of participants | 3 | Located on lines 90 - 95 of the manuscript: “Among the initial 1,077 patients, 357 were excluded based on specific criteria: (1) estimated glomerular filtration rate (eGFR) < 15 mL/min/1.73 m² (n = 31); (2) presence of autoimmune diseases (n = 14); (3) secondary IgAN conditions, such as hepatitis virus - related glomerulonephritis and allergic purpura nephritis (n = 96); (4) acute infectious diseases or cancer (n = 14); (5) incomplete or missing study data (n = 202). Ultimately, 720 eligible IgAN patients were included in the study (Fig. S1).” Although this part does not directly elaborate on the inclusion criteria, it implies them through the exclusion criteria. |
|  |  | (*b*) *Cohort study*—For matched studies, give matching criteria and number of exposed and unexposed  *Case-control study*—For matched studies, give matching criteria and the number of controls per case | Not applicable | This study is not a matched study. |
| Variables | 7 | Clearly define all outcomes, exposures, predictors, potential confounders, and effect modifiers. Give diagnostic criteria, if applicable | 3-4 | Lines 97 - 112 of this manuscript elaborate in detail on the collection of clinical and laboratory data, covering various clinical indicators and laboratory test data relevant to the study. Lines 113 - 117 of the manuscript introduce the methods and indicators for renal pathological assessment. Lines 119 - 128 of the manuscript introduce the diagnostic definition of the outcome event AKI: “Acute kidney injury (AKI)is defined according to the Kidney Disease(Kellum & Lameire 2013): Improving Global Outcomes (KDIGO) criteria: an increase in SCr of ≥ 0.3 mg/dl (≥ 26.5 μmol/l) within 48 hours; an increase in SCr of ≥ 1.5 times baseline, known or presumed to have occurred within the past 7 days; or a urine output of < 0.5 ml/kg/h for more than 6 hours. Due to the difficulty in obtaining urine output measurements, AKI is diagnosed based on changes in SCr levels in this study. For patients with AKI, all data were collected both before and after the onset of the condition. Each patient was hospitalized at least once between 2010 and 2020, with the first hospitalization during this period defined as the index admission. Since AKI may begin to develop prior to the index admission, the baseline SCr assessment period was extended to include outpatient values obtained before hospitalization.” Lines 133 - 150 introduce the definitions and calculation methods of other composite indicators, namely Systemic immune - inflammation index (SII), Systemic inflammation response index (SIRI), The pan - immune inflammation value (PIV), lymphocyte to C - reactive protein ratio (LCR), neutrophil to lymphocyte ratio (NLR), lymphocyte to monocyte ratio (LMR), platelet to lymphocyte ratio (PLR), neutrophil to high - density lipoprotein cholesterol ratio (NHR), monocyte to high - density lipoprotein cholesterol ratio (MHR), lymphocyte to high - density lipoprotein cholesterol ratio (LHR), platelet to high - density lipoprotein cholesterol ratio (PHR), The prognostic nutritional index (PNI), The geriatric nutritional risk index (GNRI). |
| Data sources/ measurement | 8* | For each variable of interest, give sources of data and details of methods of assessment (measurement). Describe comparability of assessment methods if there is more than one group | 4 | Lines 124 - 128 of the manuscript indicate the time period for data collection required for the diagnosis of AKI:” For patients with AKI, all data were collected both before and after the onset of the condition. Each patient was hospitalized at least once between 2010 and 2020, with the first hospitalization during this period defined as the index admission. Since AKI may begin to develop prior to the index admission, the baseline SCr assessment period was extended to include outpatient values obtained before hospitalization.” |
| Bias | 9 | Describe any efforts to address potential sources of bias | 5 | Located on lines 179 - 181 of the manuscript: “To enhance the reliability of the assessment, both calibration curves and DCA were performed with 1000 iterations using the bootstrap method.” |
| Study size | 10 | Explain how the study size was arrived at | 3 | Located on lines 90 - 95 of the manuscript: “Among the initial 1,077 patients, 357 were excluded based on specific criteria: (1) estimated glomerular filtration rate (eGFR) < 15 mL/min/1.73 m² (n = 31); (2) presence of autoimmune diseases (n = 14); (3) secondary IgAN conditions, such as hepatitis virus-related glomerulonephritis and allergic purpura nephritis (n = 96); (4) acute infectious diseases or cancer (n = 14); (5) incomplete or missing study data (n = 202). Ultimately, 720 eligible IgAN patients were included in the study (Fig. S1).” |

Continued on next page

| Quantitative variables | 11 | Explain how quantitative variables were handled in the analyses. If applicable, describe which groupings were chosen and why | 3 and 5 | Located on lines 99 - 100 of the manuscript: “body mass index [BMI, categorized into underweight (< 18.5), normal (18.5 to 24.9), overweight (25 to 29.9), and obese (≥ 30)]”.  Located on lines 157 – 159 of the manuscript: “Continuous variables were described using median (interquartile range). Categorical variables were expressed as percentages.” |
| --- | --- | --- | --- | --- |
| Statistical methods | 12 | (*a*) Describe all statistical methods, including those used to control for confounding | 5 | Lines 154 - 183 of the manuscript describe in detail all statistical methods. |
|  |  | (*b*) Describe any methods used to examine subgroups and interactions | Not applicable | There were no methods used to examine subgroups and interactions in this study. |
|  |  | (*c*) Explain how missing data were addressed | 3 | During the inclusion and exclusion phase of the study, for the handling of missing data, we adopted a deletion strategy. Specifically, detailed explanations can be found in line 94 of the manuscript: "Incomplete or missing study data (n = 202)". |
|  |  | (*d*) *Cohort study*—If applicable, explain how loss to follow-up was addressed  *Case-control study*—If applicable, explain how matching of cases and controls was addressed  *Cross-sectional study*—If applicable, describe analytical methods taking account of sampling strategy | 5 | During the inclusion and exclusion phase of the study, for the handling of missing data, we adopted a deletion strategy. Specifically, detailed explanations can be found in lines 90 - 95 of the manuscript. |
|  |  | (*e*) Describe any sensitivity analyses | Not applicable | Sensitivity analysis was not performed in this study. |
| Results | | | | |
| Participants | 13* | (a) Report numbers of individuals at each stage of study—eg numbers potentially eligible, examined for eligibility, confirmed eligible, included in the study, completing follow-up, and analysed | 3 | Located on lines 90 - 95 of the manuscript: “Among the initial 1,077 patients, 357 were excluded based on specific criteria: (1) estimated glomerular filtration rate (eGFR) < 15 mL/min/1.73 m² (n = 31); (2) presence of autoimmune diseases (n = 14); (3) secondary IgAN conditions, such as hepatitis virus-related glomerulonephritis and allergic purpura nephritis (n = 96); (4) acute infectious diseases or cancer (n = 14); (5) incomplete or missing study data (n = 202). Ultimately, 720 eligible IgAN patients were included in the study (Fig. S1).” |
|  |  | (b) Give reasons for non-participation at each stage | 3 | Located on lines 90 - 95 of the manuscript: “Among the initial 1,077 patients, 357 were excluded based on specific criteria: (1) estimated glomerular filtration rate (eGFR) < 15 mL/min/1.73 m² (n = 31); (2) presence of autoimmune diseases (n = 14); (3) secondary IgAN conditions, such as hepatitis virus-related glomerulonephritis and allergic purpura nephritis (n = 96); (4) acute infectious diseases or cancer (n = 14); (5) incomplete or missing study data (n = 202). Ultimately, 720 eligible IgAN patients were included in the study (Fig. S1).” |
|  |  | (c) Consider use of a flow diagram | 3 | The flowchart for screening eligible participants can be seen in Supplementary Figure S1. |
| Descriptive data | 14* | (a) Give characteristics of study participants (eg demographic, clinical, social) and information on exposures and potential confounders | 6 | Located on lines 195 - 213 of the manuscript: “The subjects’ baseline characteristics are presented in Table 1. The median age of participants was 36.0 years, with males constituting 50% of the sample. In our study, 61 participants (8.5%) were classified as AKI. Patients with AKI had a higher proportion of males, elevated MAP, and greater prevalence of diabetes, hypertension, and NS. Laboratory results indicated that AKI patients had higher levels of SCr, BUN, uric acid, CRP, 24-hour urinary protein quantification, and urine microalbumin, along with lower levels of serum albumin, IgG, and IgM. Renal pathological showed a higher incidence of E1, T1, T2, and C2 lesions in AKI patients. Regarding immune-inflammatory indexes, AKI patients exhibited higher values of SII, SIRI, PIV, NLR, NHR, and MHR, while LCR and LMR values were lower. The PNI was lower in AKI patients, whereas the difference in GNRI between the two groups was not significant. In clinical treatment, there were no differences between the two groups in using ACEI/ARB or corticosteroids before renal biopsy. The median follow-up time of participants was 26.00 months, 94 (13.06%) experienced composite endpoint events. Of the 659 patients without AKI, 77 (11.68%) experienced composite endpoint events, while 17 (27.87%) out of 61 patients with AKI experienced these events. IgAN patients with AKI had a significantly higher probability of experiencing composite endpoint events (P < 0.001). Baseline characteristics of the training and test sets showed no statistically significant differences, as detailed in Table 2. The training set included 48 patients (9.5%) with AKI, while the test set included 13 patients (6.0%) with AKI.” |
|  |  | (b) Indicate number of participants with missing data for each variable of interest | 3 | Line 94 of the manuscript mentions “incomplete or missing study data (n = 202)” |
|  |  | (c) *Cohort study*—Summarise follow-up time (eg, average and total amount) | 6 | Line 206 of the manuscript states, “The median follow - up time of participants was 26.00 months.” |
| Outcome data | 15* | *Cohort study*—Report numbers of outcome events or summary measures over time | 6 | Line 206-209 of the manuscript states, “94 (13.06%) experienced composite endpoint events. Of the 659 patients without AKI, 77 (11.68%) experienced composite endpoint events, while 17 (27.87%) out of 61 patients with AKI experienced these events.” |
|  |  | *Case-control study—*Report numbers in each exposure category, or summary measures of exposure | Not applicable | This study is not a Case-control study. |
|  |  | *Cross-sectional study—*Report numbers of outcome events or summary measures | 6 | Line 195-197 of the manuscript states, “The median age of participants was 36.0 years, with males constituting 50% of the sample. In our study, 61 participants (8.5%) were classified as AKI.” |
| Main results | 16 | (*a*) Give unadjusted estimates and, if applicable, confounder-adjusted estimates and their precision (eg, 95% confidence interval). Make clear which confounders were adjusted for and why they were included | Not applicable | This method was not used in this study. |
|  |  | (*b*) Report category boundaries when continuous variables were categorized | 3 | Located on lines 99 - 100 of the manuscript: “body mass index [BMI, categorized into underweight (< 18.5), normal (18.5 to 24.9), overweight (25 to 29.9), and obese (≥ 30)]”. |
|  |  | (*c*) If relevant, consider translating estimates of relative risk into absolute risk for a meaningful time period | Not applicable | This method was not used in this study. |

Continued on next page

| Other analyses | 17 | Report other analyses done—eg analyses of subgroups and interactions, and sensitivity analyses | 6-7 | Lines 214 - 228 of the manuscript introduce the "Risk Factors and Prediction Models for AKI in Patients with IgA Nephropathy". Lines 229 - 252 of the manuscript present the "Validation and Comparison of Prediction Models". Lines 253 - 255 of the manuscript describe "The association between AKI and long - term prognosis in IgAN patients". |
| --- | --- | --- | --- | --- |
| Discussion | | | | |
| Key results | 18 | Summarise key results with reference to study objectives | 7-8 | Line 260-281 of the manuscript states, “We observed that, compared to IgAN patients without AKI, those with AKI have a significantly higher risk of long-term renal function decline. Therefore, it is crucial to identify IgAN patients at risk of AKI early and implement preventive measures, rather than solely treating those who have already developed AKI. However, there are no practical predictive tools available to identify individuals among IgAN patients who may progress to AKI. In present study, we identified 11 risk factors for the occurrence of AKI in patients with IgAN, which include the presence of NS, T1 in the Oxford histological score, high levels of BUN, 24h-UPRO, CRP, SIRI, and PLR, as well as low eGFR, LMR, LCR, and PNI. We used the aforementioned results to construct a comprehensive model for predicting the risk of AKI in patients with IgAN. Since CRP, SIRI, LMR, PLR, and LCR are primarily related to inflammation, these inflammatory markers were extracted and individually combined with other features to construct five new prediction models, extended the usability of the models. Additionally, we employed Lasso regression to select the risk factors, identifying eGFR, BUN, and CRP, thereby developing a more streamlined model (Model 6). Consequently, a total of seven prediction models were established, along with corresponding nomograms to quantify the risk of AKI. We also evaluated the predictive performance and calibration of each model and created decision curves to assess clinical benefits. All seven models demonstrated good predictive capabilities, with the Full Model being the best performer. All models, except Model 5, exhibited good calibration. Furthermore, we discovered that all models possessed strong clinical decision-making abilities. Ultimately, we recommend prioritizing the use of the full model for predicting the risk of AKI in IgAN patients in clinical practice. Additionally, the other models, excluding Model 5, can also be considered for clinical application.” |
| Limitations | 19 | Discuss limitations of the study, taking into account sources of potential bias or imprecision. Discuss both direction and magnitude of any potential bias | 11-12 | Line 423-434 of the manuscript states, “Firstly, being a retrospective study with a relatively small sample size, this research is limited in the capacity of identification, controlling of confounding factors, and conducting in-depth stratified analyses. Consequently, the accuracy and reliability of the model may be compromised. This study will continue expanding the sample size to improve the robustness and reliability of the models. Secondly, AKI was only diagnosed by creatinine changes in this study, which may lead to an underestimation of AKI incidence. Thirdly, our renal biopsy cohort only included M1 lesions, which may limit the generalizability of the findings to M0 patients. Finally, the model development and validation were based on clinical pathological data from a single institution, lacking external validation. This may introduce geographic or other systematic biases. Future studies will require the use of multi-center data for external validation to enhance the generalizability and broader applicability of the results.” |
| Interpretation | 20 | Give a cautious overall interpretation of results considering objectives, limitations, multiplicity of analyses, results from similar studies, and other relevant evidence | 8-11 | Lines 282 - 417 of the manuscript elaborate in detail on the results of this study, namely the content and significance of the prediction model, and also delve deeply into the possible mechanisms of occurrence. |
| Generalisability | 21 | Discuss the generalisability (external validity) of the study results | 11 | Lines 430 - 434 of the manuscript discuss the generalisability of the results. |
| Other information | |  | | |
| Funding | 22 | Give the source of funding and the role of the funders for the present study and, if applicable, for the original study on which the present article is based | supplementary information | It has been mentioned in the Funding Statement of the journal's submission system. |

*Give information separately for cases and controls in case-control studies and, if applicable, for exposed and unexposed groups in cohort and cross-sectional studies.

**Note:** An Explanation and Elaboration article discusses each checklist item and gives methodological background and published examples of transparent reporting. The STROBE checklist is best used in conjunction with this article (freely available on the Web sites of PLoS Medicine at http://www.plosmedicine.org/, Annals of Internal Medicine at http://www.annals.org/, and Epidemiology at http://www.epidem.com/). Information on the STROBE Initiative is available at www.strobe-statement.org.
